# Anaerobic Sport-Specific Tests for Taekwondo: A Narrative Review with Guidelines for the Assessment

**DOI:** 10.3390/sports12100278

**Published:** 2024-10-14

**Authors:** Gennaro Apollaro, Ibrahim Ouergui, Yarisel Quiñones Rodríguez, Rafael L. Kons, Daniele Detanico, Emerson Franchini, Piero Ruggeri, Coral Falcó, Emanuela Faelli

**Affiliations:** 1 Department of Neuroscience, Rehabilitation, Ophthalmology, Genetics and Maternal Child Health, University of Genoa, 16132 Genoa, Italy; gennaro.apollaro@edu.unige.it; 2 Centro Polifunzionale di Scienze Motorie, University of Genoa, 16132 Genoa, Italy; ruggeri@unige.it (P.R.); emanuela.faelli@unige.it (E.F.); 3 High Institute of Sport and Physical Education of Kef, University of Jendouba, Kef 7100, Tunisia; brahim.ouerghi@issepkef.u-jendouba.tn; 4 Research Unit: Sport Sciences, Health and Movement, UR22JS01, University of Jendouba, Kef 7100, Tunisia; 5 Department of Physical Education and Sports, Faculty of Education and Sport Sciences, University of Granada, 52005 Melilla, Spain; yquignones_l@ugr.es; 6 Department of Physical Education, Federal University of Bahia, Salvador 40170-110, Brazil; rafael.kons@ufba.br; 7 Biomechanics Laboratory, Center of Sports, Federal University of Santa Catarina, Florianópolis 88040-900, Brazil; d.detanico@ufsc.br; 8 Martial Arts and Combat Sports Research Group, Sport Department, School of Physical Education and Sport, University of São Paulo, São Paulo 05508-030, Brazil; efranchini@usp.br; 9 Department of Experimental Medicine, Section of Human Physiology, University of Genoa, 16132 Genoa, Italy; 10 Department of Sport, Food and Natural Sciences, Western Norway University of Applied Sciences, 5020 Bergen, Norway

**Keywords:** combat sports, performance, Olympic sports, physical tests, anaerobic training, taekwondo

## Abstract

The ATP-PCr system represents the main source of energy during high-intensity attack actions in taekwondo matches. In contrast, the glycolytic system supports the maintenance of these actions when repeated techniques are performed. Given the close relationship between anaerobic energy systems and attack activity in combat, the literature relating to the use of sport-specific test protocols for anaerobic assessment has experienced a remarkable increase. This narrative review aims to illustrate the sport-specific anaerobic tests available in taekwondo by retracing and examining development and validation process for each test. Forty-one articles published between 2014 and 2023 were selected via the MEDLINE and Google Scholar bibliographic databases. These tests are the Taekwondo Anaerobic Test and Adapted Anaerobic Kick Test (i.e., continuous mode testing); the 10 s and multiple Frequency Speed of Kick Tests; the chest and head Taekwondo Anaerobic Intermittent Kick Tests; and the Taekwondo-Specific Aerobic–Anaerobic–Agility test (i.e., intermittent mode testing). Coaches and strength and conditioning professionals can use all the tests described in taekwondo gyms as they feature short and easy-to-implement protocols for monitoring and prescribing specific anaerobic training. The guidelines in this review evaluate each test from several perspectives: basic (e.g., validity, reliability, and sensitivity), methodological (e.g., continuous or intermittent mode testing) and application (e.g., time–motion structure and performance parameters). This comprehensive approach aims to assist stakeholders in selecting the most appropriate test.

## 1. Introduction

The Golden Jubilee year of World Taekwondo (WT), celebrated on 28th May 2023 [1], was characterized by significant events that marked the Olympic combat sport of taekwondo consolidation on the world sports scene. This historic year coincided with the first ten years of the World Grand Prix (i.e., four competitions per year in which the best athletes in the world participate) [2]. In this sense, the increase in high-level competitions has been accompanied by many rule changes, to ensure that the sport aligns closely to the growing sporting and media interest [3]. Studying the time–motion structure of official matches (three rounds × 2 min/1 min recovery) in high-level athletes over time [4], it is clear that the main goal of rule changes is to alter combat rhythm by emphasizing high-intensity actions as much as possible, minimizing low-intensity actions as well as referee pauses [5,6,7,8]. In the Tokyo 2020 Olympic Games, which occurred in 2021 due to the COVID-19 pandemic, athletes performed attack periods of ~2 s (s) interposed with skipping periods of ~3 s, generating an attack/skipping (A/S) ratio of ~1:1.5 [5]. In previous official competitions, although similar attack times (~2 s) emerged, the A/S ratio was between ~1:5 and 1:8, and the match structure was modulated by the combat round [6,7,8]. Therefore, the latest data showed a markedly higher A/S ratio, resulting from an almost threefold increase in the number of attacks in a round, indicating that the rule changes were effective to induce more spectacular and dynamic actions than previously [5]. If rule changes significantly alter the activity profile of the match, the physiological responses are also likely to change [9]. Janowski et al. [10] compared the kinematic profile and physiological responses in athletes who took part in official competitions under different rules, before and after the Rio 2016 Olympic Games. In the most recent competitions, the authors found that peak heart rate (HR_peak_) reached 102 ± 0.3% of the maximum heart rate (HR_max_) determined in a graded exercise test, and the post-match blood lactate concentration ([La]) was 13.8 ± 0.4 mmol∙L^−1^ [10]. The previous literature has reported HR_peak_ values between 96 ± 5 and 97 ± 2% of HR_max_, and post-match [La] values were between 6.7 ± 2.5 and 14.0 ± 4.2 mmol∙L^−1^ [7,11,12,13,14]. The framework outlined so far supports the oft-formulated assumption that high demands are placed on both aerobic and anaerobic metabolisms to sustain the typical activity of the official match [4,9,15]. Nevertheless, the most recent literature has pointed to a further shift in combat towards high-intensity activity and hypothesized an increase in the absolute contribution of the glycolytic system, although the oxidative system is likely still predominant [5,10,16].

## 2. The Role of the Anaerobic Energy Systems in Taekwondo

**Figure 1:**
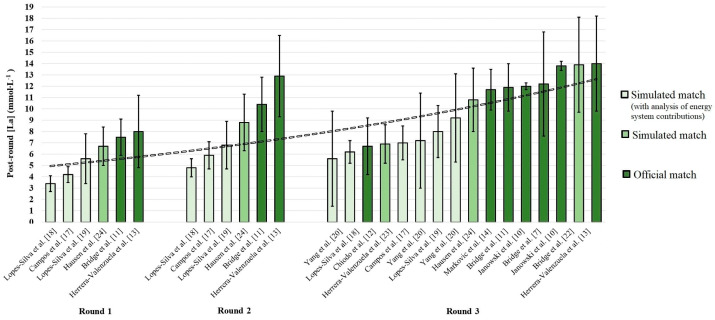
**Blood lactate values [La] post-rounds in simulated and official match**. First, some studies have quantified the [La] after each round of the match. Specific analysis of these values reveals a gradual increase in the values of [La] post-round, from round to round, with the highest value at the end of the last round of the match, in both simulated and official matches. However, data should be interpreted with caution as this increase throughout the rounds does not necessarily reflect glycolytic contribution increase [25]. Indeed, the studies [17,18,19,24] that also calculated delta (Δ) [La] (i.e., the lactate concentration after the round minus the lactate concentration at the beginning of the round) suggest a decrease in lactate accumulation and a consequent reduction in glycolytic participation throughout the rounds. Moreover, it is important to note that identifying [La]_peak_ after each round is not practicable after rounds 1 and 2 due to the short duration of recovery (i.e., 1 min) between rounds. Secondly, placing the [La] values in ascending order, for each round, shows that the values identified in simulated matches, in which the contribution of the three energy systems was quantified in parallel, are slightly lower (and with a certain degree of overlap for the values after the third round) than those found in simulated and official matches, in which only the contribution of the glycolytic system was quantified or a backpack was used to protect the portable gas analysis system. Values: mean ± SD.

The insights regarding the interaction dynamics of energy systems, as well as the predominance of the oxidative system during combat, are based on previous studies that have estimated the contribution of the three energy systems (i.e., ATP-PCr, glycolytic, oxidative) during simulated matches [17,18,19,20]. These studies showed that athletes, in order to sustain an A/S ratio between ~1:3 and 1:7, exhibited reduced reliance on anaerobic energy systems (ATP-PCr: 19–33%; glycolytic: 3–9%) and relied more on the oxidative energy system (62–74%). Overall, anaerobic energy systems were the secondary energy source utilized during the entire match and every single round (Table 1). Therefore, the assumption was formulated that the ATP-PCr system represents the main source of energy during high-intensity attack actions, while the glycolytic system supports the maintenance of these actions when repeated techniques are performed. In parallel, the oxidative system ensures the maintenance of high-intensity action, contributing to the PCr resynthesis during periods of low intensity or passive recovery [9,17,18,19,20,21]. For [La], the recorded post-simulated-match values were between 5.6 ± 4.2 and 9.2 ± 3.9 mmol∙L^−1^ [20], which were slightly lower, although overlapping (Figure 1), than those mentioned above in official matches (6.7 ± 2.5–14.0 ± 4.2 mmol∙L^−1^) [7,11,12,13,14] and those found in simulated matches (6.9 ± 1.7–13.9 ± 4.2 mmol∙L^−1^). It must be noted that, in these, only [La] was quantified at the end of rounds (i.e., the physiological indicator measured to estimate the contribution of the glycolytic system) [22,23], or a backpack was used to protect the portable gas analysis system [24]. Indeed, in the studies that quantified the three energy systems’ contribution in parallel, the positioning of the portable gas analysis system on the athlete’s back (to estimate the contribution of the oxidative and ATP-PCr systems) prevented the use of the electronic scoring system, limited the range of movements allowed to the athletes, and consequently interfered with the interactions between the athletes [17,18,19,20] (Figure 1). Thus, the different estimates and dynamics of energy system interactions hypothesized in the most recent studies [5,10] find their basis in the discrepancy between [La] values. This discrepancy is caused by the limitations of the current experimental approach to estimate the contributions of the oxidative and ATP-PCr systems, which moreover make it inapplicable to official combats [4]. In addition, the nature of combat (i.e., official or simulated) is among the main causes of these differences, as official competitions are generally characterized by higher intensity, stress, and motivation, with a consequent impact on the absolute values of [La] [7].

This experimental approach was applied in a recent study, in order to investigate the contribution of energy systems during three sport-specific high-intensity intermittent exercises (HIIEs) [25]. In this modality, the relative contribution of anaerobic energy systems was higher (42–65%), and they were the main energy source in two of the three exercises tested (51% and 54%) (Table 1), due to the specificity of each HIIE structure. Anaerobic energy systems were the predominant energy source only in the first round of all three exercises, while [La] values recorded post-protocol ranged from 6.7 ± 1.2 to 8.3 ± 0.9 mmol∙L^−1^ (Table 1). A pilot study [26] estimated the energy system’s contribution to the chest Taekwondo Anaerobic Intermittent Kick Test (TAIKT_chest_) proposed by Tayech et al. [27]. The relative anaerobic energy system’s contribution was even higher, compared to that found in specific HIIEs (Table 1), due to a greater contribution of the glycolytic system. The [La] post-test value was 9.7 ± 1.6 mmol∙L^−1^ (Table 1). Although the shorter test duration compared to a sport-specific HIIE round might influence the energy dynamics, the A/S ratio of TAIKT_chest_ is closer to the time–motion structure of the match [5]. Therefore, these data provide further insights into the anaerobic demands placed on the athlete during match activity and, even more so, into the need for scientific research to adapt anaerobic assessment methods to better reflect specific responses.

## 3. General Anaerobic Assessment in Taekwondo

Physical assessment, through the use of physical tests, aims to impact the athlete or team’s sporting performance by prescribing better training programs and, consequently, positively affect competition success [28,29]. Bridge et al. [15] found that the anaerobic fitness of taekwondo athletes was commonly assessed through the 30 s Wingate Test (WAnT) (i.e., the gold-standard methodology) [30]. In this sense, a wide range was reported for relative peak power (RPP) (6.6 ± 0.4 and 11.8 ± 2.0 W/kg) [31,32] and relative mean power (RMP) values (4.5 ± 0.6 and 9.2 ± 1.2 W/kg) [32,33] among athletes, using a load of 0.075 kp/kg of body mass. According to the authors [15], this wide range of values is caused by a variation in training status, the fiber composition of muscle groups and the motor unit activation between the different samples included in the studies. The most recent literature (Table 2) has shown that taekwondo athletes’ anaerobic fitness is still assessed through the WAnT or other general field tests such as the Running Anaerobic Sprint Test (RAST) [34], which are valid, reliable and accurate tests but lack the mechanical specificity for taekwondo actions, compromising the validity of power profiles [15]. These studies revealed RPP (7.5 ± 0.8 and 12.0 ± 1.4 W/kg) [35,36] and RMP values (5.7 ± 0.5 and 9.0 ± 0.7 W/kg) [35,36], using a load of 0.075 kp/kg of body mass, in line with previous results [15]. Nevertheless, Bridge et al. [15] had already pointed out the lack of standardization in WAnT protocols (e.g., load variation, start-up procedures, ergometric models) and the need for more specific tests that mimic both the mechanical actions and anaerobic demands of taekwondo. A decade after this insight, it is possible to state how subsequent research has been increasingly dedicated to the development of specific tests for anaerobic fitness assessments in taekwondo [37,38,39].

## 4. Sport-Specific Anaerobic Assessment in Taekwondo

In a systematic review about the methodological quality, validation data, and feasibility of specific tests for Olympic combat sports, Chaabene et al. [37] concluded that establishing valid tests that assess actual physical fitness and/or physiological attributes still remains a major concern for sports sciences. In this review, half of the taekwondo articles were about anaerobic assessment [37]. The major interest in this area of fitness could be justified by the close relationship between anaerobic energy systems and attack activity in combat [17,18,19,20]. Although in the review by Chaabene et al. [37] less than half of the articles on taekwondo addressed the assessment of endurance, Apollaro et al. [9] highlighted a remarkable increase in the literature on this topic in recent years, to the point of formulating guidelines for assessment. In this sense, considering the major research interest already found for anaerobic fitness in taekwondo and the intensification of the match that has emerged in recent studies [5,38,51], the literature regarding the development, validation, and use of sport-specific test protocols for the anaerobic assessment in taekwondo, has also experienced a consequent and remarkable increase in recent years. Thus, the objective of this narrative review is to illustrate the sport-specific tests available in the current literature for the anaerobic assessment in taekwondo, retracing and examining the development and validation process of each methodology. The methodology of narrative reviews was used to provide practical applications, recommendations, and future perspectives as guidelines for scientists, coaches, and strength and conditioning professionals. The guidelines formulated are also supported by the authors’ direct and practical experience with the topic of this narrative review. These tests are the Taekwondo Anaerobic Test (TAT) [38,51] and the Adapted Anaerobic Kick Test (AAKT) [48] (i.e., continuous mode testing); the 10 s and multiple Frequency Speed of Kick Tests (FSKT_10s_ and FSKT_mult_) [56]; the chest and head Taekwondo Anaerobic Intermittent Kick Tests (TAIKT_chest_ and TAIKT_head_) [27,57]; and the Taekwondo-Specific Aerobic–Anaerobic–Agility (TAAA) test [39] (i.e., intermittent mode testing).

### 4.1. Taekwondo Anaerobic Test (TAT), Adapted Anaerobic Kick Test (AAKT)

**Figure 2:**
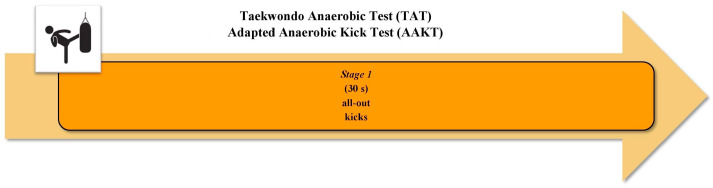
**Anaerobic continuous sport-specific tests. Taekwondo Anaerobic Test (TAT)** [38,51]. Athlete performs the bandal-chagi by alternating the legs, beginning with dominant leg, as many times as possible at maximal intensity over 30 s. Kicks must be carried out in height between the umbilical scar and xiphoid process of the athlete, marked by placing a taekwondo body protector on the punching bag. The kicking cycle is defined as the time interval between two consecutive kicks with the same leg. From this parameter, the number of kicking cycles (only completed cycles), mean kicking time and best kicking time are calculated. In addition, by measuring the magnitude of impact in each kick, the highest kicking impact and the mean kicking impact over the 30 s of the test are identified. The fatigue index (FI) is calculated using the mean kicking time and mean impact of the initial 20% cycles and the mean of the last 20% cycles [38]. During the test, the amount of performed techniques are recorded, as well as the kicking impact force. Consequently, the following performance indicators are calculated: Peak power observed during the first 5 s of the test; relative peak power; mean anaerobic power during the 30 s of the test; relative mean anaerobic power; fatigue index; and anaerobic capacity [51]. **Adapted Anaerobic Kick Test (AAKT)** [48]. Athlete performs the bandal-chagi with the dominant leg as many times as possible at maximal intensity over 30 s. The test is performed using a target pad positioned at the height of the iliac crest of the athlete. The first kick is performed with the dominant leg in the back. Starting from the second kick, the preferred leg is positioned forward throughout the remaining time of the test. Only kicks performed with the front leg are considered for the analysis. The following parameters are calculated: higher kick frequency performed during 3 s; lower kick frequency performed during 3 s; average kick frequency performed during 30 s; fatigue index (percentage reduction of the maximum frequency kick to minimum frequency kick); and time to higher kick frequency performed during 3 s.

Sant’Ana et al. [38] developed the TAT (Figure 2). The duration and kick frequency of the test were defined based on the WAnT [30]. A tri-axial accelerometer, with a preamplifier for each axis, was placed on the athletes’ dominant ankle. The highest impact peak generated by the leg with the accelerometer was used to measure the time spent for each kicking cycle. In addition, the magnitude of impact in each kick was also identified. A significant increase (11%) in kicking cycle time and a significant decrease (28%) in the magnitude of kicking impact emerged, comparing values from the first 20% to the last 20% of kicking cycles. The authors found a significant positive correlation between [La]_peak_ and the number of kicking cycles (*r* = 0.65), suggesting that a higher glycolytic energy release is related to the ability to execute more kicks during this test. In addition, a significant positive correlation between countermovement jump (CMJ) test performance and the number of kicking cycles (*r* = 0.70), and negative correlations between CMJ and mean kicking time (*r* = −0.79) and best kicking time (*r* = −0.89), were found. This indicates that the ability to perform a high-speed kick and to repeat the movement over a certain period depends on the athletes’ muscle power level. The authors concluded that the analysis of only the temporal variables and the amount of kicking cycles could allow the test to be performed with simple instrumentation, therefore improving the cost-effectiveness [38]. Rocha et al. [51] investigated the concurrent criterion validity of the TAT with the WAnT [30], as well as its test–retest reliability, by analyzing other performance parameters (Figure 2). A piezo sensor in the center of the strike shield was used to assess the impact force in each kick. All TAT performances were significantly and positively correlated with those of the WAnT (*r* = 0.55–0.88), thus showing a level of agreement, except for anaerobic capacity. In fact, a difference of approximately 22% in anaerobic capacity between the two tests was found. The difference observed in this variable could be due to a reduction in technical performance during the TAT and the consequent execution of techniques in the less sensitive areas of the protective shield [51]. Significant positive correlations emerged between CMJ and the number of techniques (*r* = 0.59) and mean anaerobic power (*r* = 0.56) during the TAT, respectively. In line with Sant’Ana et al. [38], these results suggested that the ability to perform the TAT depends on maximal muscular power production by the knee extensors. Relative test–retest reliability analysis for TAT performance revealed intraclass correlation coefficient (ICC) values between 0.80 and 0.99, showing the good to excellent reliability of the test. Finally, although the main findings led Rocha et al. [51] to attribute specificity to the TAT for anaerobic assessment, the need has emerged to use a power assessment tool able to maintain such a level of accuracy in future studies, ensuring the recording of every possible type and magnitude of hit.

Oliveira et al. [48] proposed the AAKT (Figure 2), a test with the same duration as the TAT but with a different mode of execution. A contact sensor was coupled in the target pad with an armored inertial sensor inside to measure the kick time. The authors [48] studied the concurrent criterion validity of the AAKT with the WAnT [30]. The WAnT performance, including the RPP, RMP, and fatigue index (FI), showed significant positive correlations with higher kick frequency (*r* = 0.85), average kick frequency (*r* = 0.87), and the FI (*r* = 0.86) of the AAKT, respectively. Despite these results, the authors suggested the need for further studies to re-investigate the relationship between general and sport-specific tests [48].

### 4.2. Frequency Speed of Kick Tests (FSKT_10s_ and FSKT_mult_)

**Figure 3:**
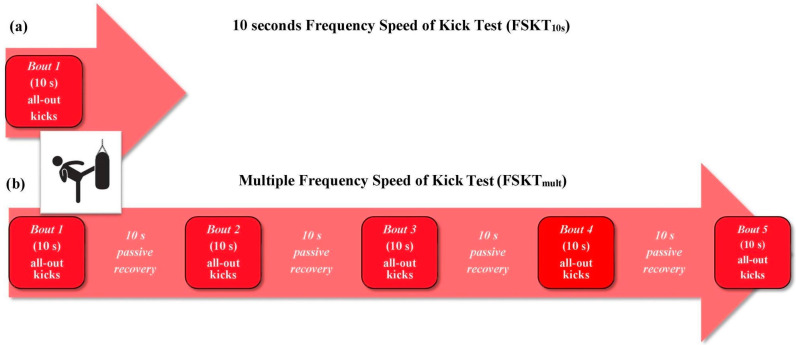
**Anaerobic intermittent sport-specific tests.** (**a**) **10 s Frequency Speed of Kick Test (FSKT_10s_)** [56]. The FSKT_10s_ lasts for 10 s. After the sound signal, athlete must execute the maximum number of bandal-chagi movements possible by alternating right and left legs. In order to accomplish the test, each athlete is placed in front of the stand bag equipped with a taekwondo body protector, positioned at the same height of the athlete trunk. The performance is determined by the total number of kicks applied during the test. (**b**) **Multiple Frequency Speed of Kick Test (FSKT_mult_)** [56]. The FSKT_mult_ consists of five 10 s sets with a 10 s passive recovery between sets. The execution criteria for the FSKT_mult_ are the same as those defined for the FSKT_10s_. The performance is determined by the number of kicks in each set, total number of kicks and kick decrement index (KDI) during the test. The KDI indicates performance decreases during the test. To calculate the KDI, the following Equation is used, which takes into account the number of kicks applied during all sets of the FSKT_mult_: KDI (%) = [1 − (FSKT_1_ + FSKT_2_ + FSKT_3_ + FSKT_4_ + FSKT_5_)/best FSKT × number of sets] × 100. **Video Analysis**. Both tests are recorded, and the videos are analyzed posteriorly to manually count the kicks performed, through video analysis software. First, the count starts when the athlete moves the attack feet and finishes when he touches the bag. Valid kicks are those that hit the target during 10 s. If the athlete starts the kick before completing 10 s but reaches the target only after 10 s, the kick is not considered valid. Second, the valid kicks are those performed with appropriate technique and power.

Initially proposed by Villani et al. [58,59] for kickboxing, Santos et al. [60,61] adapted an additional sport-specific test called the FSKT (Figure 3) for taekwondo. The FSKT was developed into two versions (FSKT_10s_ and FSKT_mult_). The insight of these authors was to point out that the continuous application of the TAT and AAKT could reduce the ecological validity of the assessment, as the taekwondo match is intermittent in nature [60,61]. The FSKT_10s_ consists of performing the greatest number of bandal-chagi movements in the most powerful way possible in 10 s, while the FSKT_mult_ consists of performing five sets of the FSKT_10s_ with 10 s of passive recovery in between. However, only the test–retest reliability of the FSKT_mult_ [58] was reported, limiting the accuracy of the results [60,61]. In this sense, Santos and Franchini [62] investigated the sensitivity of the FSKT to nine weeks of taekwondo-specific training. The results showed that athletes significantly improved test performance after the training period, with a higher improvement than the smallest worthwhile change (SWC), suggesting that the FSKT is sensitive to the short training period. The SWC_0.2_ was 0.28 for the FSKT_10s_ and between 0.26 and 1.77 for the FSKT_mult_. The authors concluded that the performance parameters of the FSKT_10s_ and FSKT_mult_ can be used to estimate maximum anaerobic power and the ability to repeat sport-specific high-intensity intermittent efforts, respectively [62].

Santos and Franchini [63] examined the discriminant construct validity of the FSKT by comparing performance parameters between international-/national- and state-/regional-level female athletes. International-/national-level female athletes presented significantly higher performance than their state/regional counterparts, with the exception of the FSKT_4_, the FSKT_5_, and the kick decrement index (KDI), which did not differ between the groups. The authors suggested that the FSKT can be used to indirectly assess the sport-specific anaerobic performance of female taekwondo athletes and is able to adequately discriminate female athletes of different competitive levels [63]. Santos et al. [64] established two classificatory tables for the FSKT, for female as well as male athletes. Additionally, the authors compared FSKT performance among Olympic weight categories, in order to determine whether the classification could be generalized among athletes. There were no significant differences in any FSKT performance among female athletes of different weight categories. In contrast, FSKT_4_ performance differed among the male weight categories, as athletes in the feather category achieved better performance than those in the middle category. Also, the KDI was smaller for the fly category than the feather category. Based on the developed classificatory tables (see Santos et al. [64]), the authors concluded that it is possible to monitor taekwondo sport-specific anaerobic fitness easily and cost-effectively among athletes of different Olympic weight categories using the FSKT. However, due to the significant differences found, caution should be exercised when investigating male athletes of different weight categories [64].

Santos et al. [56] analyzed the discriminant construct validity of the FSKT by comparing performance among international/national and state/regional male athletes, and non-competitors. International-/national-level male athletes performed significantly more kicks than non-competitors during the FSKT_10s_, FSKT_3_, FSKT_5_ and FSKT_total_. The authors also studied this aspect of validity between international/national and state/regional male athletes through receiver operating characteristic (ROC) curve analysis [56]. The area under the ROC curve was between 0.51 and 0.60 for the FSKT performance. Overall, these results indicated a low ability of the FSKT to discriminate between international/national and state/regional male athletes. Therefore, this test characteristic was not confirmed among male athletes, contrasting to what was found in female athletes, although ROC curves were not used in that case [63]. According to Santos et al. [56], the grouping at the international/national or state/regional levels eliminated possible differences between the groups. They suggested the importance of future studies with separate groups (e.g., international vs. national) or among different levels of experience (e.g., novice vs. advanced athletes) to investigate the discriminant construct validity of the FSKT [56].

Another aim of Santos et al. [56] was to establish the test–retest reliability of the FSKT. The ICC revealed a relative reliability value of 0.95 for the FSKT_10s_, and values ranged between 0.63 and 0.83 for FSKT_mult_ performance, thus showing a moderate to excellent reliability. The coefficient of variation (CV) recorded an absolute reliability value of 2.9% for the FSKT_10s_ and values ranging from 4.1 to 20.4% for FSKT_mult_ performance. The only CV value above the indicated limit of 5% was that of the KDI (20.4%), as this parameter considers all series of the FSKT_mult_ and the possible changes that may occur in each one. Other studies have also reported test–retest reliability values for the FSKT, but only of the FSKT_total_ for the FSKT_mult_. Specifically, the FSKT_10s_ and FSKT_total_ presented ICCs of 0.76–0.95 and 0.85–0.77, respectively [60,63,64,65,66,67,68,69,70,71,72,73,74], and CVs of 2.9% and 3.9%, respectively [60,63,64,65,67], showing data that supported the test–retest reliability of the FSKT. Antonaccio et al. [75] established the inter-/intra-rater reliability of the FSKT. The ICC revealed a relative reliability value of 1.00 for the FSKT_10s_, and values between 0.99 and 1.00 for FSKT_mult_ performance, thus indicating excellent reliability. The CV recorded an absolute reliability value of 0% for both the FSKT_10s_ and FSKT_mult_. Other studies have also reported high relative intra-rater reliability values for the FSKT_10s_ (ICC = 1.00) and FSKT_mult_ (ICC = 0.99–1.00) [56,62], in line with the results of Antonaccio et al. [75]. In addition, Santos et al. [56] investigated the sensitivity of the FSKT to identify the minimum individual change required for them to be confident that a real change had occurred. The SWC_0.6_ was 1.43 for the FSKT_10s_ and ranged from 0.73 to 4.83 for FSKT_mult_ performance. Therefore, the FSKT was considered a suitable test for detecting small variations due to its higher values for the SWC_0.6_ than the typical error of measurement (TEM). The minimal detectable change (MDC_95%_) was 1.67 for the FSKT_10s_ and ranged from 2.17 to 11.07 for FSKT_mult_ performance.

Santos and Franchini [76] tested the logical translational validity of the FSKT to determine how coaches and strength and conditioning professionals with different backgrounds understand the procedures, application and energy systems contribution to FSKT. The stakeholders were divided into three groups (undergraduates, graduates, and postgraduates in physical education and sports science) and logical validity was tested with a specific questionnaire. It emerged that stakeholders considered the FSKT to be very easy to understand and endowed with a very feasible application, and that the energy systems’ contribution to the tests was predominantly anaerobic. The educational degree influenced these aspects of the test. In particular, the higher response frequency for the anaerobic prevalence of the FSKT_10s_ and FSKT_mult_ was evident as education levels increased. However, future studies are needed to estimate the energy systems’ contribution of taekwondo athletes to the FSKT and confirm or refute this assumption. In conclusion, Santos and Franchini [76] stated that the FSKT is a test that has logical validity for the assessment of sport-specific anaerobic fitness, in line with what was previously reported [56,60,63]. However, the professional degree of the stakeholder could have a major impact on the test chosen for the anaerobic assessment.

Ribeiro et al. [77] stated that the available tests (e.g., TAT, FSKT) consist of performing the maximum number of kicks in a fixed period of time. According to the authors, this characteristic of the tests could be considered a limitation because the number of kicks is not an accurate unit of measurement for detecting small improvements in performance. They hypothesized that changing the analysis of the number of kicks performed in a fixed period of time to the time it takes (in milliseconds) to perform a fixed number of kicks could give the FSKT greater accuracy [77]. Therefore, Ribeiro et al. [77] developed a hardware and software system that was able to identify 100% of the kicks performed, measure the time to perform the fixed number of 20 kicks, and control the pause time between the five sets of 10 s. The time recorded to perform 20 kicks ranged from 9.4 to 11.0 s, in line with the time structure of FSKT. In addition, the time increased index (TII) (i.e., the KDI for the FSKT_mult_) showed the typical decrease in performance along the sets, and the high values of the rating of perceived exertion (RPE) confirmed the intense nature of the test. These results suggested that the modifications made to the FSKT did not alter the physical and physiological demands of the test, had few feasibility implications, and gave the test greater accuracy. The authors also established the test–retest reliability and sensitivity of the system [77]. The ICC revealed relative reliability values ranging from 0.60 to 0.83 for performance, showing moderate to good reliability. In contrast, the ICC of the TII was 0.41 and classified as poor. The TEM recorded absolute reliability values ranging from 326 to 1539 ms for performance and 3% for the TII. The SWC_1.2_ was between 905 and 4278 ms for performance and 3% for the TII. Therefore, the system presented higher values for the SWC_1.2_ than the TEM. The MDC_95%_ was between 905 and 4266 ms for performance and 3% for the TII. However, Ribeiro et al. [77] concluded that the need for specific equipment and software to perform the test could limit its practical use. Thus, the possibility of using a video analysis-based method as a valid alternative was also assessed. This analysis showed high agreement and significant positive correlations between the time recorded by the system and that found by video analysis (*r* = 0.98–1.00), suggesting that using a camera and video analysis software could facilitate test use [77].

### 4.3. Taekwondo Anaerobic Intermittent Kick Tests (TAIKT_chest_ and TAIKT_head_)

**Figure 4:**
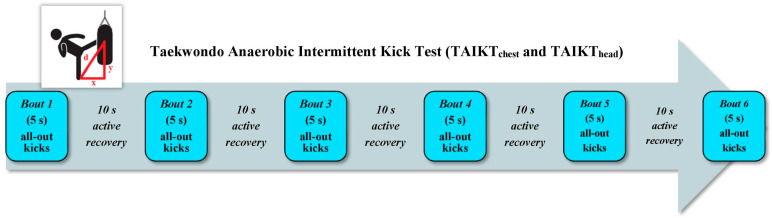
**Anaerobic intermittent sport-specific tests. Taekwondo Anaerobic Intermittent Kick Test (TAIKT_chest_)** [27]. The TAIKT_chest_ consists of six 5 s sets with a 10 s active recovery (i.e., very light [tempo = one bounce/s] bouncing movements controlled by an evaluator) between sets. After the sound signal, athlete must execute the maximum number of bandal-chagi movements possible by alternating right and left legs. In order to accomplish the test, each athlete is placed in front of the stand bag equipped with a taekwondo electronic body protector, positioned at the same height of the athlete trunk, i.e., at a height (y) relative to the mat. During kick execution, the athlete should not exceed a mark on the mat, the optimum distance (x) to be determined before the test, to effectively execute kicking on the body protector. The distances (x) and (y) allow to determine the distance (d) using the Pythagorean Theorem, which is the projection distance of the foot on the body protector. Participants are asked to wear their official protectors during the test. The number of kicks is automatically displayed on the computer screen after each kicking set and the scoring threshold is set according to the criteria used in the competition for each weight category. TAIKT_chest_ performance is expressed as absolute (W) and relative (W·kg^−0.67^) peak power (P_peakTAIKT_) and mean power (P_meanTAIKT_), and absolute (W) fatigue index (FI_TAIKT_). P_peakTAIKT_ is the highest power output of the six sets of kicks; P_meanTAIKT_ is sum of powers of six sets of kicks/6; FI_TAIKT_ is P_peakTAIKT_-minimum power (P_minTAIKT_)/total test duration (30 s). The authors have made an Excel spreadsheet available in which performance can be calculated by entering the known values, i.e., body mass, x and y distances, and number of kicks in each series. **Taekwondo Anaerobic Intermittent Kick Test (TAIKT_head_)** [57]. The execution criteria and performance for the TAIKT_head_ are the same as those defined for the TAIKT_chest_, except that in the TAIKT_head_, the kicks are projected on a dummy’s head covered by an electronic head protector.

Tayech et al. [27] highlighted the limitations of the sport-specific tests presented thus far. Among others, the active phases exceed the average time of attack sequences (e.g., TAT and FSKT), and the time structure does not reflect the average A/S ratio of the match (e.g., FSKT_mult_). In addition, the validity of kicks is charged to the evaluator (e.g., FSKT). Therefore, they developed the TAIKT_chest_ (Figure 4), which consists of scoring the greatest number of bandal-chagi movements delivered to a bag covered by an electronic body protector during six sets of 5 s interposed with 10 s of active recovery [27]. Tayech et al. [27] studied the concurrent criterion validity of the test with the RAST [34]. Positive significant correlations emerged between the performance (*r* = 0.60–0.81) and physiological responses (HR_peak_, [La]_peak_, and RPE; *r* = 0.55–0.89) of the two tests. It is interesting to note that TAIKT_chest_ performance was also expressed relatively using allometric expression (W·kg^−0.67^) since body mass is a criterion for ranking taekwondo athletes. The physiological responses confirmed the importance of glycolytic system activation and the high intensity of the TAIKT_chest_ [27]. Tayech et al. [27] also established the test–retest reliability and sensitivity of the TAIKT_chest_. The ICC revealed relative reliability values ranging from 0.68 to 0.99 for performance and physiological responses, showing moderate to excellent reliability. The TEM recorded absolute reliability values ranging from 0.01 to 5.14 for performance and physiological responses. The SWC_0.2_ was between 0.05 and 1.83 for performance and physiological responses. Therefore, the TAIKT_chest_ presented higher values for the SWC_0.2_ than the TEM, except for HR_peak_ and RPE. The MDC_95%_ was between 0.02 and 14.26 for performance and physiological responses.

Tayech et al. [55] analyzed the concurrent criterion validity of the TAIKT_chest_ with the WAnT [30], as well as the relationship between the sport-specific test, the squat jump (SJ), and CMJ (i.e., tests used to assess lower-limb anaerobic power). Positive significant correlations emerged (*r* = 0.47–0.66) between TAIKT_chest_ and WAnT performance, except for RMP. The physiological responses (HR_peak_, [La]_peak_, and RPE) of the two tests also showed positive significant correlations (*r* = 0.53–0.55), suggesting an important contribution of the glycolytic system to support the physical demands of the TAIKT_chest_. On the other hand, positive significant correlations emerged between the RPP of the TAIKT_chest_, expressed allometrically, and that of SJ (*r* = 0.60) and CMJ (*r* = 0.63). Thus, the TAIKT_chest_ is highly dependent on explosive lower-limb actions such as those used in taekwondo competitions [55]. Tayech et al. [55] also investigated the discriminant construct validity of the TAIKT_chest_ by comparing performance and physiological responses between elite and sub-elite athletes [55]. Elite athletes expressed significantly more power than sub-elite athletes in all TAIKT_chest_ performances, while no difference emerged for physiological responses [55]. In addition, ROC curve analysis revealed an area under the curve between 0.79 and 0.96 for TAIKT_chest_ performance. These results indicated a high ability of the TAIKT_chest_ to discriminate athletes of different performance levels [55]. Overall, Tayech et al. [27,55] stated that the TAIKT_chest_ is a non-invasive, easy-to-perform and accessible test. In addition, the intermittent nature and physiological responses of the TAIKT_chest_ are very close to those of competitions.

Boutios et al. [41] also studied the concurrent criterion validity of the TAIKT_chest_ with the WAnT [30]. Their analysis revealed slightly lower correlations between the performance (*r* = 0.06–0.41) and responses of [La]_peak_ (*r* = 0.43) in the TAIKT_chest_ and WAnT, compared with those reported by Tayech et al. [55]. According to the authors, the different physical demands imposed by the two tests could have influenced the assessments of anaerobic performance. Specifically, although it uses a sport-specific technique, the TAIKT_chest_ requires optimal technical preparation, which could be influenced by the athletes’ level. The higher degree of correlation between the TAIKT_chest_ and WAnT, shown in the study of Tayech et al. [55], could be related to the higher level of the recruited athletes, compared to those of Boutios et al. [41]. Würdig et al. [26] investigated the energy systems’ contribution to the TAIKT_chest_, aiming to provide relevant information for its use as a method to assess anaerobic fitness. The results confirmed the predominance of anaerobic energy systems in providing energy during the TAIKT_chest_, with a contribution of ~70% (ATP-PCr: ~48%; glycolytic: ~23%). These findings, when combined with the results of the other aspects of validity, reliability, and sensitivity [27,41,55], strengthened the usefulness of the TAIKT_chest_ for the control and monitoring of the anaerobic component in taekwondo athletes.

Tayech et al. [57] hypothesized that the adaptation of the TAIKT_chest_ based on further technological advancement (i.e., the electronic helmet protector, and modifications to the scoring system) would further improve the assessment of intermittent high-intensity physical performance. To this aim, they developed the TAIKT_head_ (Figure 4). This test has the same structure and performance as the TAIKT_chest_ but the kicks are projected to the head of the dummy, covered by an electronic helmet protector [57]. First, Tayech et al. [57] determined the concurrent criterion validity of the TAIKT_head_ with the 30 s continuous jump test (CJ) and investigated the relationship between the sport-specific test, CMJ and flexibility performance. Positive significant correlations emerged between TAIKT_head_ and CJ performance (*r* = 0.64–0.84) and physiological responses (*r* = 0.43–0.74). On the other hand, positive significant correlations between the APP and RPP of the TAIKT_head_ and CMJ (*r* = 0.88 and 0.79, respectively) were reported, while no relationship emerged in flexibility performance. Second, the authors assessed the convergent construct validity between the two versions of the TAIKT [57]. Positive significant correlations emerged between performance (*r* = 0.53–0.74) and physiological responses (*r* = 0.60–0.72) in both tests. In particular, the highest HR_peak_ and [La]_peak_ values indicated the maximum intensity of the TAIKT_head_ and are in line with those reported in competitions. Additionally, aiming to test the interchangeability of the two methods, linear regression was used to model the relationship between performance. This analysis showed that TAIKT_head_ performance was partially predicted by that of the TAIKT_chest_ and vice versa [57]. Third, Tayech et al. [57] investigated the discriminant construct validity of the TAIKT_head_ by comparing performance and physiological responses between elite and sub-elite athletes. Elite athletes expressed significantly more power than sub-elite athletes in all performances, while no differences emerged for physiological responses. ROC curve analysis revealed an area under the curve between 0.71 and 0.85 for TAIKT_head_ performance. These results indicated a high ability of the TAIKT_head_ to discriminate athletes of different performance levels [57]. Finally, a further aim was to establish the test–retest reliability and sensitivity of the TAIKT_head_ [57]. The ICC revealed relative reliability values between 0.87 and 0.99 for performance and physiological responses, showing good to excellent reliability. The TEM recorded absolute reliability values between 0.01 and 1.48 for performance and physiological responses. The SWC_0.2_ was between 0.04 and 1.64 for performance and physiological responses. Therefore, the TAIKT_head_ presented higher values for the SWC_0.2_ than TEM, except for the RPE. The MDC_95%_ was between 0.02 and 4.10 for performance and physiological responses [57].

### 4.4. Taekwondo-Specific Aerobic–Anaerobic–Agility (TAAA) Test

**Figure 5:**
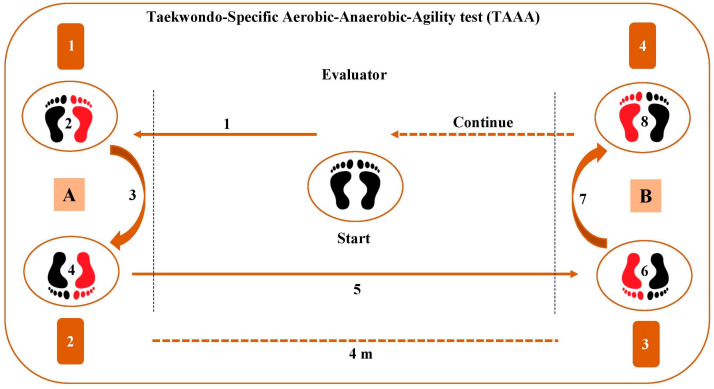
**Anaerobic intermittent sport-specific test. Taekwondo-Specific Aerobic–Anaerobic–Agility (TAAA) test** [39]. The test involves six 20 s (a total of 2 min) intervals of shuttle sprints over a 4 m distance, and the execution of the bandal-chagi to the punching bags alternating the legs at the end of each distance, with 10 s rest intervals between the sets. For a detailed description of this test, refer to the previous review on sport-specific assessment of endurance in taekwondo [9]. To estimate anaerobic fitness are calculated: maximum kicks (maximum number of kicks in a 20 s interval); minimum kicks (minimum number of kicks in a 20 s interval); average kicks (total number of kicks at the end of the test divided by six) and kick fatigue index (KFI) according to following Equation: KFI (%) = [Maximum Kicks − Minimum Kicks)/Total Kicks] × 100.

Taati et al. [39] reported that tests that were previously developed (e.g., TAT, FSKT_mult_, TAIKT) are characterized by execution times that do not reproduce the typical duration of a match round, in addition to the stationary nature of the tests [27,38,56]. Therefore, they developed the TAAA test (Figure 5) utilizing an intermittent nature (with six 20 s of sprints and kicks, interposed with 10 s of rest intervals), including turns and movements across set distances [39]. This test was previously presented in the review on the sport-specific assessment of endurance in taekwondo [9]. The inclusion of this methodology, also in the present review, is more than justified as it is the only proposed test, so far, to estimate multiple important components of fitness in taekwondo. Focusing our interest on the anaerobic assessment, the authors investigated the concurrent criterion validity of the TAAA test with the WAnT [30] in order to indirectly determine the sport-specific anaerobic fitness of athletes. The number of kicks performed in the TAAA test was classified from poor to excellent. The average CV for the TAAA test and the WAnT were 11.7% and 9.3%, respectively. Positive significant correlations emerged between RMP and RPP, obtained from the WAnT, and average (*r* = 0.79) and maximum (*r* = 0.54) kicks, during the TAAA test, respectively. The authors developed a linear regression model to estimate average power values according to the relationship between average power output obtained in the WAnT and average kicks recorded in the TAAA test. The following Equation (1) could explain 63% of the overall variability between the variables, with a standard error of estimate (SEE) of 0.53 W·kg^−1^.

Average Power (W·kg^−1^) = 0.648 (AK), (1)

where AK is the total number of kicks at the end of the TAAA test divided by six.

In parallel, the authors [39] examined the discriminant construct validity through the ROC curve’s analysis. The area under the ROC curve was 0.74, 0.75, and 0.72, for maximum, average, and minimum kicks, respectively. These results indicated a very good ability of the test to discriminate regional- and national-level taekwondo athletes. A performance, equation, and classification table (see Taati et al. [39]) of the TAAA test could offer a convenient and cost- and time-efficient way to monitor and classify sport-specific anaerobic fitness. On the other hand, the lack of [La] measurement, the standardization of the type of displacement, and the measurement of the impact of kicks represent areas for future investigations [39].

## 5. Practical Applications

The sport-specific tests presented could be used to study the acute effects of post-activation potentiation (PAP), ergogenic supplements, or music on anaerobic performance in athletes of different age groups (cadets, juniors, and seniors) and competitive levels (Regional, state, national and international). For example, Santos et al. [61] investigated the effects of different rest intervals and conditioning activities on lower-limb power and FSKT_10s_ performance, aiming to identify the positive PAP effect to maximize performance. Pak et al. [78] analyzed the effects of two mouth-rinsing solutions (glucose and caffeine) plus a placebo condition on sport-specific anaerobic performance during the Ramadan period. The TAIKT_chest_ was performed after supplementation before, during, and after Ramadan weeks to monitor test performance. All the analyzed sport-specific tests could be used to study the chronic effects of general and sport-specific training protocols on anaerobic performance, both during the preparatory and competitive periods. For example, Santos and Franchini [62] investigated the effects of nine weeks of taekwondo and strength and conditioning training on FSKT performance, executed in the pre- and post-training weeks. Aravena Tapia et al. [65] compared the effects of four weeks of an additional sport-specific high-intensity interval training (HIIT) program and a control situation on FSKT_mult_ performance. Both groups executed the test before and after the training period. The performance of all sport-specific anaerobic tests could be used to investigate their relationship to general and sport-specific performance related to fitness components (such as endurance and agility), fundamental competition requirements (such as lower-limb power) or body composition characteristics. For example, Ojeda-Aravena et al. [79] investigated the relationship between body composition characteristics, lower-limb power, and physical responses to anaerobic, general and specific endurance, and agility tests. Albuquerque et al. [80] established the relationship between lower-limb power and physical and physiological responses to sport-specific anaerobic and endurance tests. In these studies, sport-specific anaerobic performance was assessed using the FSKT_10s_ and/or the FSKT_mult_. The sport-specific tests presented could be used to determine fundamental anaerobic parameters that are useful for subsequent training prescription, although no studies have used them for this purpose in practice. For example, Franchini [81] indicated that the determination of supramaximal performance parameters, during the execution of the FSKT_10s_ or first series of the FSKT_mult_, and maximal performance parameters, derived from sport-specific tests for endurance such as the PSTT (see Apollaro et al. [9]), could be useful for prescribing short HIIT intervals.

The main performance variables and physiological responses measured during the execution of the FSKT and TAIKT in the validation and practical application studies are shown in Table 3 and Table 4, respectively.

## 6. Discussion

The presentation of all available tests for sport-specific anaerobic assessment in taekwondo and their practical applications highlights that each has specific methodological and application characteristics that must be considered when choosing which test to utilize [9]. The TAT [38,51] and AAKT [48] represent the first proposed taekwondo-specific anaerobic tests. These tests share the time structure inspired by the WAnT (30 s); thus, the continuous application does not mimic the mean time of a high-intensity action (~2 s) and the intermittence of the match. Although these tests were the first attempts at sport-specific assessment, they are also the least-studied tests, and we have no evidence of practical applications due to the requirement of expensive instrumentations. The FSKT [56] and TAIKT [27,57] are the most-studied and utilized tests. The FSKT_mult_ has been given an intermittent nature but it is necessary to consider that the active phases of the test (10 s) far exceed the average time of the attack sequences (~2 s), and the time structure (1:1) does not reflect the typical average A/S ratio of the match (~1:1.5 and 1:8) [5,6,7,8]. In addition, the analysis of physiological responses to the two versions of the FSKT (HR, [La], and RPE) has been neglected in both validation and practical application studies, representing an important area of investigation for future studies. The TAIKT is overall closer to the actual taekwondo match regarding active phase duration (5 s) and time structure (1:2). In fact, we previously mentioned that in the Tokyo 2020 Olympic Games, athletes performed attack periods of ~2 s (s), generating an A/S ratio of ~1:1.5 [5]. However, taekwondo is characterized by systematic rule changes, and further evidence on the physical and physiological demands of athletes during more recent matches will likely provide further support for the ecological validity of the TAIKT. The authors of the TAIKT developed a method to express performance in terms of absolute and relative power so that the athletes’ body mass is also considered in the evaluation. While the validity of kicks in the FSKT is the responsibility of the evaluator, the electronic devices in the TAIKT are used to quantify valid kicks objectively but increase the cost of evaluation. In both cases, the percentage of successful kicks (calculated by also taking invalid kicks into account) is not expected in the performance, although it has been calculated in some studies [78,90]. This parameter should be included to expand the insights derived from tests because the anaerobic assessment in these tests is strongly influenced by the technical component and the power of the kick. The TAAA test [39] has execution times that mimic the duration of a match round (2 min) and provides a predictive equation for estimating average power output, although physiological responses should be investigated in future studies.

Validity is a basic criterion for a physical test to be considered applicable [29,37]. Apollaro et al. [9], reviewing the literature relative to sport-specific endurance assessment in taekwondo, reported that content translational validity [29,37] was defined for all tests by referring to the specific literature. In our review, we found that this aspect was established in the same way for all tests. In addition, Würdig et al. [26] estimated the contribution of energy systems to the TAIKT_chest_ to clarify and strengthen the content validity also through an experimental approach. On the other hand, logic translational validity was tested only for the FSKT, representing the only attempt to investigate this aspect. Concurrent criterion validity [29,37] was defined for all taekwondo sport-specific endurance tests by comparing the new proposals with classical incremental treadmill testing protocols and/or field tests [9]. Similarly, this aspect has been determined for most sport-specific anaerobic tests by comparing their performance and/or physiological responses with those of gold-standard (e.g., WAnT or CJ) and/or field tests (e.g., RAST). In contrast, no study has tested the concurrent criterion validity of the FSKT, representing a necessary area for future investigations, considering its wide use. Criterion predictive validity [29,37] was not established for any taekwondo sport-specific endurance test [9], nor for any anaerobic tests presented in our review. While this shortcoming negatively impacts the validity of sport-specific tests in taekwondo, it may highlight a certain degree of difficulty in structuring experimental designs for studying this aspect. Discriminant construct validity [29,37] was neglected for most taekwondo sport-specific endurance tests, while for some tests, it was analyzed by recruiting small samples and/or using a statistical approach not also based on ROC curve analysis. In this regard, the authors [9] focused on the importance of size and heterogeneity of the samples, as the number of recruited participants in the validation and practical application studies ranged from 7 to 46, while regarding sex, there were 319 males and 26 females overall. We found that this aspect was analyzed for the FSKT and TAAA test by comparing male or female athletes at different competitive levels (international/national, state/regional, non-competitive), whereas for the TAIKT, it was tested by comparing elite and sub-elite athletes, also using ROC curve analysis. Additionally, the number of participants in the studies included in our review exhibited a wide range, varying from 8 to 153, with a total of 802 males and 291 females overall. Apollaro et al. [9] found two approaches to determining construct convergent validity [29,37] among taekwondo sport-specific endurance tests. In our review, the comparison between the TAIKT_chest_ and TAIKT_head_ represents the only attempt to study this aspect of validity. In agreement with the authors [9], we suggest that the current availability of multiple sport-specific methods should be accompanied by the analysis of their interchangeability to further encourage their use.

A physical test should also be reliable, ensure minimal noise (i.e., variability in performance), and be sensitive enough to detect improvements in physical quality when they occur [29]. In the current review, we found that test–retest reliability [29,37] was determined for the TAT, FSKT and TAIKT. Apollaro et al. [9] reported that this aspect was established for only one of the sport-specific endurance tests. They stated that the variation in stepping intensity could negatively impact the reliability of the test [9]. In this sense, while the TAIKT provides active recoveries with stepping between sets, the FSKT_mult_ uses passive recovery between sets. Inter-/intra-rater reliability [29,37] has not been studied for any sport-specific endurance test, representing an important shortcoming, since, in most tests, the evaluator is responsible for the visual inspection of each kick [9]. Regarding sport-specific anaerobic assessment, this aspect was established for the FSKT because the kicks that were considered valid in this test are the responsibility of the evaluator. Instead, in the TAIKT, electronic protections (body or helmet protector) are used to quantify valid kicks objectively. Sensitivity [29,37] has not been established for any taekwondo sport-specific endurance test [9]. In contrast, this additional basic criterion for a physical test has been investigated for the FSKT by finding a greater improvement in test performance than the SWC after a nine-week training period. In parallel, other studies have only statistically compared the SWC and TEM and calculated the MDC_95%_ for FSKT and TAIKT performance.

Finally, with the aim of simply and cost-effectively monitoring sport-specific anaerobic performance, Santos et al. [64] and Taati et al. [39] developed the first classification tables for the FSKT and the TAAA test, respectively. This tool should be developed for all presented tests considering the important practical utility and, most importantly, it should be specific to each weight and age category.

## 7. Summary

The ATP-PCr system represents the main source of energy during high-intensity attack actions in taekwondo matches. In contrast, the glycolytic system supports the maintenance of these actions when repeated techniques are performed. Considering the close relationship between anaerobic energy systems and attack activity in combat, the literature regarding the development, validation and use of sport-specific test protocols for anaerobic assessment in taekwondo has experienced a consequent and remarkable increase in recent years. These tests are the TAT and the AAKT, the FSKT_10s_ and FSKT_mult_, the TAIKT_chest_ and TAIKT_head_, and the TAAA test. The TAT and AAKT share the time structure inspired by the WAnT; thus, the continuous application does not mimic the mean time of a high-intensity action and the intermittence of the match. The FSKT_mult_ has been given an intermittent nature but it is necessary to consider that the active phases of the test exceed the average time of the attack sequences, and the time structure does not reflect the typical average A/S ratio of the match. The TAIKT is closer to a match regarding active phase duration and time structure. The TAAA test has execution times that mimic the duration of a match round. Coaches and strength and conditioning professionals can use all the tests described in taekwondo gyms as they feature short and easy-to-implement protocols for monitoring and prescribing specific anaerobic training. The guidelines in this review evaluate each test from several perspectives: basic (e.g., validity, reliability, and sensitivity), methodological (e.g., continuous or intermittent mode testing) and application (e.g., time–motion structure and performance parameters). This comprehensive approach aims to assist stakeholders in selecting the most appropriate test.

## Figures and Tables

**Table 1:** Contribution of the anaerobic energy systems and blood lactate concentration during simulated match, training, and test in taekwondo athletes.

Study	Athlete Characteristics (n°, Sex, Country)	Exercise (Duration)	Round	ATP-PCr Energy System		Glycolytic Energy System		[La] (mmol·L^−1^)
				Absolute (kJ)	Relative (%)	Absolute (kJ)	Relative (%)	
Bartel et al. (2022) [25]	NR (9 Brazilian males)	Training protocol (3 rounds × 2 min/ 1 min recovery)						
		15:10:5	Round 1	79 ± 28	50 ± 5	20 ± 6	13 ± 4	5.8 ± 1.7
			Round 2	77 ± 29	43 ± 7	8 ± 5	5 ± 2	7.6 ± 1.6
			Round 3	75 ± 28	41 ± 7	4 ± 3	2 ± 1	8.3 ± 0.9
			Total	232 ± 81	44 ± 5	32 ± 7	7 ± 1	NR
		100%TKDtest	Round 1	79 ± 16	50 ± 6	11 ± 3	7 ± 2	3.8 ± 1.0
			Round 2	80 ± 14	42 ± 3	7 ± 3	3 ± 1	5.4 ± 1.2
			Round 3	74 ± 22	39 ± 6	6 ± 3	3 ± 1	6.7 ± 1.2
			Total	232 ± 49	43 ± 4	23 ± 6	4 ± 1	NR
		35:5	Round 1	87 ± 27	58 ± 8	11 ± 4	7 ± 2	3.6 ± 1.2
			Round 2	85 ± 32	44 ± 7	10 ± 6	5 ± 3	6.0 ± 2.3
			Round 3	80 ± 19	44 ± 4	4 ± 6	2 ± 3	6.7 ± 2.4
			Total	253 ± 72	49 ± 5	25 ± 10	5 ± 2	NR
Campos et al. (2012) [17]	National and international (10 Brazilian males)	Simulated match (3 rounds × 2 min/ 1 min recovery)						
			Round 1	49 ± 11	31 ± 7	11 ± 4	7 ± 2	4.2 ± 0.7
			Round 2	49 ± 10	26 ± 5	7 ± 4	4 ± 2	5.9 ± 1.2
			Round 3	63 ± 32	30 ± 12	6 ± 5	3 ± 3	7.0 ± 1.5
			Total	54 ± 21	30 ± 6	8 ± 5	4 ± 2	5.7 ± 1.6
Lopes-Silva et al. (2018) [18]	National (9 Brazilian males)	Simulated match (3 rounds × 2 min/ 1 min recovery)						
			Round 1	40 ± 11 a	25 ± 4 a	9 ± 3 a	6 ± 2 a	3.4 ± 0.7 a
			Round 2	41 ± 12 a	25 ± 4 a	6 ± 2 a	4 ± 1 a	4.8 ± 0.8 a
			Round 3	50 ± 14 a	28 ± 5 a	6 ± 4 a	4 ± 2 a	6.2 ± 1.0 a
			Total	43 ± 10 a	26 ± 2 a	7 ± 1 a	5 ± 1 a	NR
Lopes-Silva et al. (2015) [19]	National and international (10 Brazilian males)	Simulated match (3 rounds × 2 min/ 1 min recovery)						
			Round 1	43 ± 7 a	27 ± 3 a	13 ± 7 a	9 ± 5 a	5.6 ± 2.2 a
			Round 2	43 ± 9 a	26 ± 2 a	8 ± 6 a	4 ± 3 a	6.8 ± 2.1 a
			Round 3	55 ± 24 a	33 ± 9 a	6 ± 3 a	3 ± 2 a	8.0 ± 2.3 a
			Total	NR	NR	NR	NR	NR
Würdig et al. (2023) [26]	NR (5 Brazilian males)	Test protocol (80 s)						
		TAIKT_chest_	Total	72 ± 25	48 ± 9	34 ± 9	23 ± 3	9.7 ± 1.6
Yang et al. (2018) [20]	Regional (5 German males)	Simulated match (3 rounds × 2 min/ 1 min recovery)						
			Total	19 ± 4 b	19 ± 3 b	10 ± 6 b	9 ± 4 b	9.2 ± 3.9 b¶
			Total	20 ± 3 b	20 ± 4 b	8 ± 6 b	7 ± 4 b	7.2 ± 4.2 b¶
			Total	20 ± 2 b	21 ± 3 b	6 ± 5 b	5 ± 4 b	5.6 ± 4.2 b¶

Note: 15:10:5 = 15 s of observation: 10 s of preparation (five dollyo-chagi [roundhouse kick to the helmet gear] with a controlled frequency of one kick every 2 s): 5 s of interaction (all-out dollyo-chagi) × four effort blocks; 100%TKD test = dollyo-chagi, at maximal frequency kick identified in the Continuous Taekwondo-Specific Test [9], continuously throughout each round; 35:5 = 35 s of observation and preparation: 5 s of interaction (all-out dollyo-chagi) × three 40 s effort blocks; TAIKT_chest_ = six 5 s sets (all-out bandal-chagi [roundhouse kick to the chest]) with a 10 s active recovery between sets. [La] = Post-round blood lactate concentration. a = Placebo condition values; b = Control condition values; ¶ = After the third round; NR = Not reported. Values are expressed as mean ± SD.

**Table 2:** Performance variables during the execution of general anaerobic tests in taekwondo athletes.

Study	Athlete Characteristics (n°, Sex, Country)	Test Details	P_peak_ (W)	P_peak_ (W/kg)	P_mean_ (W)	P_mean_ (W/kg)
Alp and Gorur (2020) [40]	International (Korean, 5 males, 5 females)	30 s Wingate test (Load: 0.075 kp/kg of body mass)	644.8 ± 161.1	9.3 ± 2.4	469.5 ± 127.3	6.5 ± 1.3
Boutios et al. (2022) [41]	NR (Greek, 5 males, 10 females)	30 s Wingate test (Load: 0.075 kp/kg of body mass)	596.4 ± 157.2	NR	469.5 ± 113.2	NR
Chacón-Torrealba et al. (2020) [42]	National (18 Belgian males and females)	30 s Wingate test (Load: 0.075 kp/kg of body mass)	718.5 ± 139.8 *a 728.3 ± 146.6 *a	9.9 ± 1.5 *a 9.9 ± 1.9 *a	579.7 ± 107.9 *a 564.1 ± 109.1 *a	7.9 ± 1.1 *a 7.7 ± 1.3 *a
Ju-Sik (2019) [43]	National and international (8 NR)	30 s Wingate test (Load: NR kp/kg of body mass)	495.1 ± 131.5	NR	364.9 ± 111.9	NR
Jung et al. (2018) [44]	NR (Korean, 21 males, 14 females)	30 s Wingate test (Load: 0.075 kp/kg of body mass)	NR	10.4 ± 0.2 *a 10.6 ± 0.3 *a	505.1 ± 18.1 *a 494.7 ± 20.9 *a	NR
Khayyat et al. (2020) [35]	International (12 Turkish males)	30 s Wingate test (Load: 0.075 kp/kg of body mass)	893.1 ± 105.0	12.0 ± 1.4	673.8 ± 51.8	9.0 ± 0.7
Khazaei et al. (2023) [45]	National and international (17 Iranian females)	30 s Wingate test (Load: 0.075 kp/kg of body mass)	NR	8.8 ± 1.3 a 8.2 ± 1.3 a	NR	6.2 ± 0.5 a 6.0 ± 0.8 a
Kwon et al. (2019) [46]	NR (20 Korean males)	30 s Wingate test (Load: 0.075 kp/kg of body mass)	751.2 ± 122.0	10.2 ± 0.6	633.7 ± 84.3	8.6 ± 0.5
Nabilpour et al. (2023) [47]	National and international (10 Iranian males)	30 s Wingate test (Load: 0.075 kp/kg of body mass)	572.1 ± 8.3	11.8 ± 1.1	NR	NR
Oliveira et al. (2015) [48]	National and international (Brazilian, 10 males, 5 females)	30 s Wingate test (Load: 0.075 kp/kg of body mass)	649.7 ± 92.4	10.3 ± 1.3	546.8 ± 80.1	8.8 ± 0.9
Ozan and Kiliç (2018) [49]	National and international (10 Turkish males)	30 s Wingate test (Load: 0.075 kp/kg of body mass)	748.5 ± 136.4	10.5 ± 1.2	571.1 ± 112.95	NR
Rhyu and Cho (2014) [50]	NR (20 Korean males)	30 s Wingate test (Load: NR kp/kg of body mass)	NR	9.6 ± 0.5 a 9.1 ± 0.8 a	NR	7.9 ± 0.3 a 7.6 ± 0.6 a
Rocha et al. (2016) [51]	National and international (17 Portuguese males)	30 s Wingate test (Load: 0.075 kp/kg of body mass)	663.8 ± 89.3 c	10.7 ± 1.3 c	470.6 ± 75.1 c	7.6 ± 0.9 c
Seo et al. (2019) [52]	National (47 Korean males)	30 s Wingate test (Load: 0.075 kp/kg of body mass)	686.9 ± 124.4 a 702.0 ± 143.1 a 697.8 ± 60.6 a 709.4 ± 100.1 a	10.4 ± 0.7 a 10.6 ± 0.5 a 10.7 ± 0.5 a 10.6 ± 0.8 a	492.3 ± 38.8 a 502.9 ± 94.4 a 492.3 ± 46.1 a 501.5 ± 60.4 a	7.6 ± 0.5 a 7.6 ± 0.6 a 7.5 ± 0.4 a 7.6 ± 0.7 a
Seo et al. (2019) [53]	National (47 Korean males)	30 s Wingate test (Load: 0.075 kp/kg of body mass)	620.8 ± 75.8 715.4 ± 117.9 689.9 ± 81.8	10.1 ± 0.6 10.6 ± 0.9 10.7 ± 0.5	455.1 ± 38.9 502.1 ± 60.7 502.5 ± 69.4	7.5 ± 0.4 7.5 ± 0.8 7.8 ± 0.4
Sun et al. (2022) [54]	National and international (Chinese, 6 males, 4 females)	30 s Wingate test (Load: 0.090 kp/kg of body mass)	1006.0 ± 366.8 b	14.6 ± 3.5 b	527.3 ± 152.7 b	7.7 ± 1.2 b
Taati et al. (2022) [39]	Regional and national (48 Iranian males)	30 s Wingate test (Load: 0.075 kp/kg of body mass)	NR	NR	NR	7.3 ± 0.7
Taskin and Akkoyunlu (2016) [36]	National and international (14 Turkish females)	30 s Wingate test (Load: 0.075 kp/kg of body mass)	442.4 ± 74.5	7.5 ± 0.8	337.2 ± 48.2	5.7 ± 0.5
Tayech et al. (2019) [27]	National and international (Tunisian, 7 males, 2 females)	Running Anaerobic Sprint Test (RAST)	541.4 ± 191.8	34.0 ± 9.1 ¶	439.3 ± 141.7	27.7 ± 6.7 ¶
Tayech et al. (2020) [55]	National and international (Tunisian, 14 males, 4 females)	30 s Wingate test (Load: 0.075 kp/kg of body mass)	623.0 ± 188.6	39.0 ± 9.0 ¶	443.7 ± 122.9	27.8 ± 5.7 ¶

P_peak_ (W): Peak power; P_peak_ (W/kg): Relative peak power; P_mean_ (W): Mean power; P_mean_ (W/kg): Relative mean power. a = Pre-intervention values; b = Placebo condition values; c = test–retest values; ¶ = W·kg^−0.67^; NR = Not reported. Values are expressed as mean ± SD. * = mean ± SEM.

**Table 3:** Performance variables and physiological responses measured during the execution of the 10 s Frequency Speed of Kick Test (FSKT_10s_) and the Multiple Frequency Speed of Kick Test (FSKT_mult_) in taekwondo athletes.

Study	Athlete Characteristics (n°, Sex, Country)	FSKT_10s_ (n° Kicks)	FSKT_mult_							HR_peak_ (b·min^−1^)	[La]_peak_ (mmol·l^−1^)	RPE (a.u.)
			FSKT_1_ (n° Kicks)	FSKT_2_ (n° Kicks)	FSKT_3_ (n° Kicks)	FSKT_4_ (n° Kicks)	FSKT_5_ (n° Kicks)	FSKT_total_ (n° Kicks)	KDI (%)			
Albuquerque et al. (2022) [80]	National and international (Brazilian, 29 males, 13 females)	NR	24 ± 3	23 ± 2	23 ± 4	21 ± 2	21 ± 3	112 ± 12	9.1 ± 4.2	NR	NR	NR
Antonaccio et al. (2022) [75]	Regional, state, national and international (14 Brazilian males)	21 ± 2	21 ± 3	20 ± 2	19 ± 2	18 ± 2	18 ± 2	97 ± 10	8.0 ± 3.0	NR	NR	NR
Aravena Tapia et al. (2020) [65]	National (Chilean, 10 males, 2 females)	NR	18 ± 2 a 18 ± 4 ac	17 ± 1 a 18 ± 3 ac	16 ± 1 a 17 ± 3 ac	16 ± 2 a 17 ± 3 ac	15 ± 2 a 16 ± 4 ac	81 ± 8 a 86 ± 16 ac	7.7 ± 4.9 a 7.6 ± 2.5 ac	NR	NR	NR
Castro-Garrida et al. (2020) [82]	National (8 Chilean males) Novices Advanced	NR	18 ± 1 c 21 ± 2 c	16 ± 2 c 18 ± 1 c	15 ± 2 c 17 ± 2 c	14 ± 2 c 16 ± 2 c	13 ± 2 c 16 ± 1 c	75 ± 6 c 87 ± 7 c	RF c RF c	NR	NR	NR
Chacón-Torrealba et al. (2020) [42]	National (18 Belgian males and females)	RF a RF a	13 ± 5 a 10 ± 5 a	13 ± 4 a 12 ± 3 a	12 ± 5 a 13 ± 3 a	13 ± 4 a 11 ± 4 a	11 ± 5 a 10 ± 5 a	61 ± 21 a 56 ± 17 a	17.3 ± 9.9 a 19.3 ± 14.4 a	154 ± 25 a 155 ± 24 a	15.3 ± 3.1 a 13.4 ± 3.9 a	NR
Chen et al. (2021) [66]	National (15 Taiwanese males)	NR	23 ± 2 c	22 ± 2 c	21 ± 2 c	20 ± 2 c	19 ± 2 c	105 ± 8 c	9.5 ± 5.0 c	173 ± 12 c	NR	6.8 ± 1.1 c†
Chiu et al. (2022) [83]	State and national (13 Taiwanese males)	NP	28 ± 7 ac	25 ± 6 ac	24 ± 6 ac	24 ± 5 ac	24 ± 5 ac	107 ± 51 ac	RF ac	NR	NR	NR
Santos and Franchini (2018) [63]	Regional, state, national and international (42 Brazilian females) Regional and state National and international	19 (17–20) NP 20 (19–21) NP	19 (18–20) NP 20 (19–21) NP	18 (17–19) NP 19 (18–20) NP	17 (16–18) NP 18 (17–19) NP	16 (16–17) NP 17 (16–18) NP	16 (15–17) NP 17 (16–18) NP	86 (82–90) NP 91 (86–96) NP	8.4 (4.6–10.3) NP 9.5 (5.6–11.0) NP	NR	NR	NR
Santos and Franchini (2016) [62]	State, national and international (Brazilian, 4 males, 4 females)	20 ± 1 a	20 ± 1 a	19 ± 2 a	18 ± 2 a	17 ± 2 a	17 ± 2 a	90 ± 9 a	7.6 ± 3.2 a	NR	NR	NR
Santos et al. (2019) [64]	Recreational, regional, state, national and international (Brazilian, 115 males, 70 females) −58 kg male −68 kg male −80 kg male +80 kg male −49 kg female −57 kg female −67 kg female +67 kg female	20 (19–22) NP 20 (18–22) NP 19 (18–21) NP 19 (19–21) NP 18 (17–20) NP 19 (18–20) NP 19 (17–20) NP 18 (17–20) NP	21 (19–23) NP 19 (19–22) NP 20 (18–21) NP 19 (18–21) NP 18 (17–20) NP 19 (18–20) NP 19 (17–20) NP 19 (18–20) NP	19 (18–21) NP 19 (18–21) NP 19 (18–20) NP 18 (17–20) NP 18 (17–19) NP 18 (18–19) NP 18 (17–20) NP 18 (17–19) NP	19 (18–20) NP 18 (18–20) NP 18 (17–19) NP 18 (17–19) NP 17 (16–18) NP 18 (17–18) NP 17 (16–18) NP 17 (16–18) NP	18 (17–19) NP 18 (17–19) NP 17 (16–18) NP 17 (16–18) NP 17 (15–18) NP 17 (16–18) NP 17 (16–18) NP 16 (15–17) NP	17 (16–19) NP 17 (16–19) NP 16 (16–18) NP 16 (16–18) NP 16 (15–17) NP 17 (16–17) NP 16 (16–17) NP 15 (15–16) NP	93 (88–101) NP 91 (87–100) NP 89 (85–95) NP 88 (83–95) NP 85 (82–90) NP 88 (82–90) NP 87 (82–93) NP 85 (80–89) NP	9.5 (8.1–12.2) NP 7.1 (4.5–10.3) NP 8.6 (6.0–11.7) NP 7.1 (5.5–12.1) NP 6.4 (5.1–8.3) NP 8.3 (3.2–10.5) NP 7.8 (3.5–9.5) NP 10.0 (3.4–11.1) NP	NR	NR	NR
Santos et al. (2016) [60]	State, national and international (9 Brazilian males)	NR	RF	RF	RF	RF	RF	82 ± 9 c	19.2 ± 7.9 c	NR	NR	NR
Santos et al. (2020) [56]	Regional, state, national and international (14 Brazilian males) Non-competitors, regional, state, national and international (153 Brazilian males) Non-competitors Regional and state National and international	21 ± 2 19 (18–20) NP 20 (19–21) NP 20 (19–21) NP	21 ± 2 19 (18–20) NP 19 (18–21) NP 20 (19–22) NP	20 ± 2 18 (17–20) NP 19 (17–20) NP 19 (18–21) NP	19 ± 2 17 (16–19) NP 18 (17–19) NP 19 (17–20) NP	18 ± 1 17 (16–18) NP 17 (16–19) NP 18 (16–19) NP	18 ± 1 16 (15–17) NP 17 (16–18) NP 17 (16–18) NP	97 ± 8 87 (82–94) NP 90 (85–96) NP 93 (86–96) NP	8.3 ± 3.1 8.0 (5.7–10.6) NP 7.8 (5.0–11.0) NP 8.3 (6.0–12.0) NP	NR	NR	NR
Santos et al. (2018) [67]	Regional, state, national and international (16 Brazilian males)	20 ± 3	21 ± 2	20 ± 2	19 ± 2	18 ± 2	18 ± 2	95 ± 9	7.5 ± 3.6	NR	NR	NR
Santos et al. (2015) [61]	State, national and international (11 Brazilian males)	19 ± 3 c	NR	NR	NR	NR	NR	NR	NR	NR	NR	NR
Delleli et al. (2023) [68]	National and international (16 Tunisian males)	26 ± 1 c	NR	NR	NR	NR	NR	125 ± 2 c	NR	NR	NR	9.6 ± 0.5 c†
Mesquita et al. (2019) [84]	National and international (Brazilian, 12 males, 7 females)	NR	RF b	RF b	RF b	RF b	RF b	RF b	RF b	NR	NR	RF b †
Miraftabi et al. (2021) [85]	National (8 Iranian males)	NP	RF b RF c	RF b RF c	RF b RF c	RF b RF c	RF b RF c	RF b RF c	RF b RF c	190 ± 5 b 190 ± 8 c	13.4 ± 3.0 b 12.1 ± 2.9 c	15.0 ± 2.0 b¶ 13.0 ± 2.0 c¶
Ojeda-Aravena et al. (2020) [79]	Regional and national (14 Chilean males and females)	NR	NR	NR	NR	NR	NR	96 ± 7	9.5 ± 3.3	NR	NR	NR
Ojeda-Aravena et al. (2021) [86]	National (Chilean, 11 males, 5 females)	NP	NP	NP	NP	NP	NP	96 ± 8 a 95 ± 7 ac	11.1 ± 4.3 a 7.9 ± 3.9 ac	NR	NR	NR
Ojeda-Aravena et al. (2021) [87]	National and international (Chilean, 8 males, 4 females)	NR	NR	NR	NR	NR	NR	93 ± 10 a 93 ± 10 a	6.1 ± 2.5 a 3.4 ± 2.4 a	NR	NR	NR
Ojeda-Aravena et al. (2023) [88]	National and international (Chilean, 8 males, 9 females)	NR	19 ± 2	18 ± 2	18 ± 2	17 ± 2	17 ± 2	91 ± 10	4.8 ± 3.4	NR	NR	NR
Orellana-Lepe et al. (2023) [89]	National (10 Chilean males)	NR	18 ± 2 c	18 ± 2 c	18 ± 2 c	18 ± 2 c	17 ± 2 c	88 ± 9 c	6.5 ± 4.1 c	NR	NR	NR
Ouergui et al. (2023) [69]	Regional, national and international (Tunisian, 26 males, 26 females)	25 ± 1 b 24 ± 2 b 25 ± 1 c 23 ± 2 c	NR	NR	NR	NR	NR	124 ± 2 b 111 ± 9 b 123 ± 2 c 101 ± 10 c	NR	NR	NR	NR
Ouergui et al. (2022) [70]	Regional and national (Tunisian, 14 males, 13 females)	20 ± 2 c	21 ± 2 c	19 ± 2 c	19 ± 1 c	18 ± 1 c	17 ± 1 c	95 ± 6 c	8.9 ± 3.5 c	NR	NR	NR
Ouergui et al. (2023) [71]	Regional and national (Tunisian, 13 males, 8 females)	20 ± 1 c	22 ± 1 c	20 ± 1 c	20 ± 1 c	19 ± 1 c	18 ± 1 c	99 ± 6 c	10.0 ± 4.0 c	NR	NR	NR
Ouergui et al. (2023) [72]	Regional and national (Tunisian, 10 males, 10 females)	24 ± 2 c	24 ± 2 c	22 ± 2 c	20 ± 2 c	20 ± 1 c	20 ± 2 c	106 ± 7 c	13.0 ± 2.0 c	NR	NR	7.0 (7.0–8.0) c NP †
Ouergui et al. (2023) [73]	Regional and national (Tunisian, 10 males, 10 females)	22 ± 2 c	24 ± 2 c	22 ± 3 c	21 ± 2 c	20 ± 2 c	19 ± 2 c	105 ± 9 c	13.0 ± 2.0 c	NR	NR	6.9 ± 1.0 c†
Ouergui et al. (2022) [74]	Regional and national (Tunisian, 10 males, 10 females)	23 ± 1 c	23 ± 2 c	21 ± 2 c	20 ± 2 c	19 ± 2 c	18 ± 2 c	101 ± 8 c	12.0 ± 3.0 c	NR	NR	7.4 ± 1.0 c†
Ribeiro et al. (2020) [77]	National and international (Brazilian, 15 males, 2 females)	–	9420 ± 776 ms	9719 ± 746 ms	10237 ± 754 ms	10711 ± 756 ms	11006 ± 713 ms	51094 ± 3565 ms	9.0 ± 3.0	NR	NR	10.0 ± 1.0 NP †

FSKT_10s_ = 10 s Frequency Speed of Kick Test; FSKT_mult_ = Multiple Frequency Speed of Kick Test; FSKT_1_ = Set 1 of FSKT_mult_; FSKT_2_ = Set 2 of FSKT_mult_; FSKT_3_ = Set 3 of FSKT_mult_; FSKT_4_ = Set 4 of FSKT_mult_; FSKT_5_ = Set 5 of FSKT_mult_; FSKT_total_ = Total number of kicks in the 5 sets of FSKT_mult_; KDI = Kick decrement index during FSKT_mult_. HR_peak_: Peak heart rate during the FSKT_mult_; [La]_peak_: Post-FSKT_mult_ peak blood lactate concentration; RPE: Post-FSKT_mult_ ratings of perceived exertion. a = Pre-intervention values; b = Placebo condition values; c = Control condition values; NR = Not reported; RF = Reported in figure (see the reference); ¶ = Borg RPE 6–20; † = Borg CR 0–10. Values expressed as mean ± SD. NP = Nonparametric distributed values expressed as median (interquartile range).

**Table 4:** Performance variables and physiological responses measured during the execution of the chest-marking version of the chest Taekwondo Anaerobic Intermittent Kick Test (TAIKT_chest_) and the head Taekwondo Anaerobic Intermittent Kick Test (TAIKT_head_) in taekwondo athletes.

Study	Athlete Characteristics (n°, Sex, Country)	P_peak_ (W) (W·kg^−0.67^)	P_mean_ (W) (W·kg^−0.67^)	FI (W·s^−1^)	S_kicks_ (n)	HR_peak_ (b·min^−1^)	[La]_peak_ (mmol·l^−1^)	RPE (a.u.)
Boutios et al. (2022) [41] TAIKT_chest_	National and international (Greek, 5 males, 10 females)	346.5 ± 80.2 NR	284.1 ± 63.7 NR	32.7 ± 7.0 *	NR	NR	12.6 ± 1.4	NR
Pak et al. (2020) [78] TAIKT_chest_	State (Turkish, 18 males, 9 females)	NR	NR	NR	40 ± 7 ab	NR	NR	RF ab¶
Sarshin et al. (2021) [90] TAIKT_chest_	National (40 Iranian males)	NR	NR			NR	RF	NR
								
		1.3 ± 0.2 a	1.1 ± 0.2 a	21.4 ± 6.7 a	59 ± 3 a			
		1.4 ± 0.1 a	1.2 ± 0.1 a	25.1 ± 7.4 a	61 ± 1 a			
		1.3 ± 0.2 a	1.1 ± 0.1 a	26.2 ± 9.3 a	60 ± 1 a			
		1.3 ± 0.1 ab	1.1 ± 0.2 ab	26.1 ± 5.8 ab	60 ± 2 ab			
		1.4 ± 0.1 ac	1.1 ± 0.1 ac	29.1 ± 9.4 ac	59 ± 2 ac			
Tayech et al. (2019) [27] TAIKT_chest_	National and international (Tunisian, 15 males, 5 females)	14.6 ± 6.5	9.8 ± 5.1	14.4 ± 6.3	NR	187 ± 9	10.6 ± 1.6	14.0 ± 1.4 ¶
		0.9 ± 0.3	0.6 ± 0.3					
Tayech et al. (2020) [55] TAIKT_chest_	National and international (Tunisian, 15 males, 5 females)	14.6 ± 6.2	9.7 ± 4.4	0.3 ± 0.1		189 ± 9	11.0 ± 1.6	13.8 ± 0.9 ¶
		0.9 ± 0.3	0.6 ± 0.2					
Elite		19.0 ± 5.1	13.0 ± 3.3	0.4 ± 0.1	NR	188 ± 8	11.2 ± 1.6	13.6 ± 1.1 ¶
		1.1 ± 0.2	0.8 ± 0.2					
Sub-elite		10.7 ± 3.2	6.8 ± 2.2	0.3 ± 0.1		192 ± 8	10.8 ± 1.6	14.0 ± 0.7 ¶
		0.7 ± 0.2	0.5 ± 0.2					
Tayech et al. (2022) [57] TAIKT_chest_	National and international (Tunisian, 21 males, 6 females)	11.7 ± 5.6	7.7 ± 4.5	62.1 ± 18.4 *		183 ± 9	10.5 ± 2.2	6.0 ± 1.0 †
		0.7 ± 0.3	0.5 ± 0.3					
TAIKT_head_		16.3 ± 4.7	14.5 ± 4.3	25.2 ± 4.3 *		182 ± 10	9.8 ± 2.8	5.0 ± 2.0 †
		1.0 ± 0.2	0.9 ± 0.2					
Elite		18.5 ± 4.7	16.2 ± 4.5	24.9 ± 5.4 *	NR	181 ± 12	9.7 ± 2.9	5.0 ± 2.0 †
		1.1 ± 0.2	1.0 ± 0.2					
Sub-elite		13.6 ± 2.9	12.3 ± 3.1	25.6 ± 2.2 *		184 ± 7	10.0 ± 2.8	5.0 ± 1.0 †
		0.9 ± 0.2	0.8 ± 0.2					
Würdig et al. (2023) [26] TAIKT_chest_	NR (5 Brazilian males)	21.4 ± 6.3	18.9 ± 5.1	10.5 ± 6.9	NR	187 ± 14	9.7 ± 1.6	14.4 ± 2.0 ¶
		0.5 ± 0.1	0.4 ± 0.1					

TAIKT_chest_: Chest-marking version of Taekwondo Anaerobic Intermittent Kick Test; TAIKT_head_: Head-marking version of Taekwondo Anaerobic Intermittent Kick Test; P_peak_: Peak power; P_mean_: Mean power; FI: Fatigue index; S_kicks_: Total successful kicks; HR_peak_: Peak heart rate during the test; [La]_peak_: Post-test peak blood lactate concentration; RPE: Post-test ratings of perceived exertion. a = Pre-intervention values; b = Placebo condition values; c = Control condition values; NR = Not reported; RF = Reported in figure (see the reference); * = (%); ¶ = Borg RPE 6–20; † = Borg CR 0–10. Values expressed as mean ± SD.

## Data Availability

Data analyzed during this review are available from the corresponding author upon reasonable request.
